# Efficacy of the lumbar sympathetic ganglion block in lower limb pain and its application prospects during the perioperative period

**DOI:** 10.1002/ibra.12069

**Published:** 2022-10-09

**Authors:** Jing‐Han Zhang, Yan‐Ping Deng, Min‐Jian Geng

**Affiliations:** ^1^ Department of Anesthesiology, Class 2020 Group Southwest Medical University Luzhou China; ^2^ Department of Anesthesiology Southwest Meducal University Luzhou China; ^3^ Duke University Medical Center Durham NC USA; ^4^ Department of Anesthesiology Nanchong Central Hospital Nanchong China

**Keywords:** gastroenteric function, lower limb pain, lumbar sympathetic ganglion block, postoperative analgesia

## Abstract

The sympathetic nervous system is involved in the physiological pathogenesis of many different types of chronic pain. Sympathetic blocks can interrupt the reflex control system by intercepting the noxious afferent fibers accompanying autonomic nerves, resulting in changes in peripheral or central sensory processing. A lumbar sympathetic ganglion block (LSGB), as a treatment method, refers to the injection of nerve blockers into the corresponding lumbar sympathetic nerve segments, usually requiring imaging assistance (CT, X‐ray, ultrasound) to guide. At present, LSGB has been widely used in the clinical treatment of lower limb pain, such as neuropathic pain, lower limb ischemic pain, and so on. Its mechanism of action may be through inhibiting sympathetic nerve activity and dilating blood vessels, thereby alleviating pain and inhibiting stress response. However, there are few reports of LSGB during the perioperative period, especially in postoperative pain and gastrointestinal function. Therefore, by studying the literature about LSGB‐related studies, this article reviews the anatomy of the lumbar sympathetic nerve (LSN), with its clinical application and possible mechanism. We reviewed the analgesic effect of LSGB in patients with lower limb pain and postoperative pain and the potential application prospects in the recovery of gastrointestinal function, finally providing a reference for its clinical application.

## Graphical abstract

At present, a lumbar sympathetic ganglion block (LSGB) has been widely used in the clinical treatment of lower limb pain, such as neuropathic pain, lower limb ischemic pain, and so on. Its mechanism of action may be through inhibiting sympathetic nerve activity and dilating blood vessels, thereby alleviating pain and inhibiting stress response. However, there are few reports of LSGB during the perioperative period, especially in postoperative pain and gastrointestinal function. Therefore, through the literature retrieval about LSGB‐related studies, this article reviews the anatomy of the lumbar sympathetic nerve, its clinical application, and possible mechanism, which elucidates the analgesic effect of LSGB in patients with lower limb pain and postoperative pain and the potential application prospects in the recovery of gastrointestinal function, finally providing a reference for its clinical application.

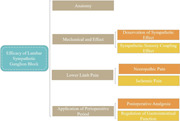

## 1 INTRODUCTION

The sympathetic nervous system (SNS) has a wide range of effects and plays a vital role in managing pain states and pathologies in the body.^1^, ^2^ Sympathetic nerves play a crucial signaling role in the pathological process of pain. Research has shown that efferent sympathetic fiber activity, in the periphery, can upregulate afferent nociceptive fiber pain signals. Mechanisms may include: on the one hand, norepinephrine, discharged by postganglionic sympathetic fibers, directly works on nociceptive fibers, which increases pain signals at any point along the nerve; on the other hand, SNS activity indirectly increases pain perception through interactions with other processes.^1^ Sympathetic‐maintaining pain (SMP) can take place in varieties of pain syndromes, for example, complex regional pain syndrome (CRPS),^3^ and pain syndromes with this characteristic are able to affect work, relationships, and mental health and even debilitating.^4^, ^5^, ^6^ SNS‐driven pain signaling can be inhibited by blocking key sympathetic nerves or ganglia.^7^, ^8^

A lumbar sympathetic ganglion block (LSGB) is widespread in the diacrisis and therapy of SMP.^9^, ^10^ An LSGB refers to injecting drugs (local anesthetic drugs: lidocaine, ropivacaine, etc.) into the lumbar sympathetic ganglia of the corresponding segment to destroy the nerve conduction function, thereby achieving the method of treating certain diseases. LSGB technology has become increasingly popular in recent decades, and multitudinous diseases are treated with LSGB, including neuropathic pain (NP), vascular pain, and pain put down to spider bites, hyperhidrosis disease, erythematous extremity pain, and so on.^11^, ^12^, ^13^, ^14^, ^15^

Although the clinical application of LSGB is becoming more and more popular, there are few reports on the application of LSGB during the perioperative period. Therefore, this article will focus on the relevant literature on LSGB for the therapeutic effect of lower limb pain and discuss its application prospect during the perioperative period and the potential application prospect in the recovery of gastrointestinal function, so as to provide experience for its clinical treatment.

## 2 ANATOMY OF THE LUMBAR SYMPATHETIC NERVE (LSN)

**Figure 1:**
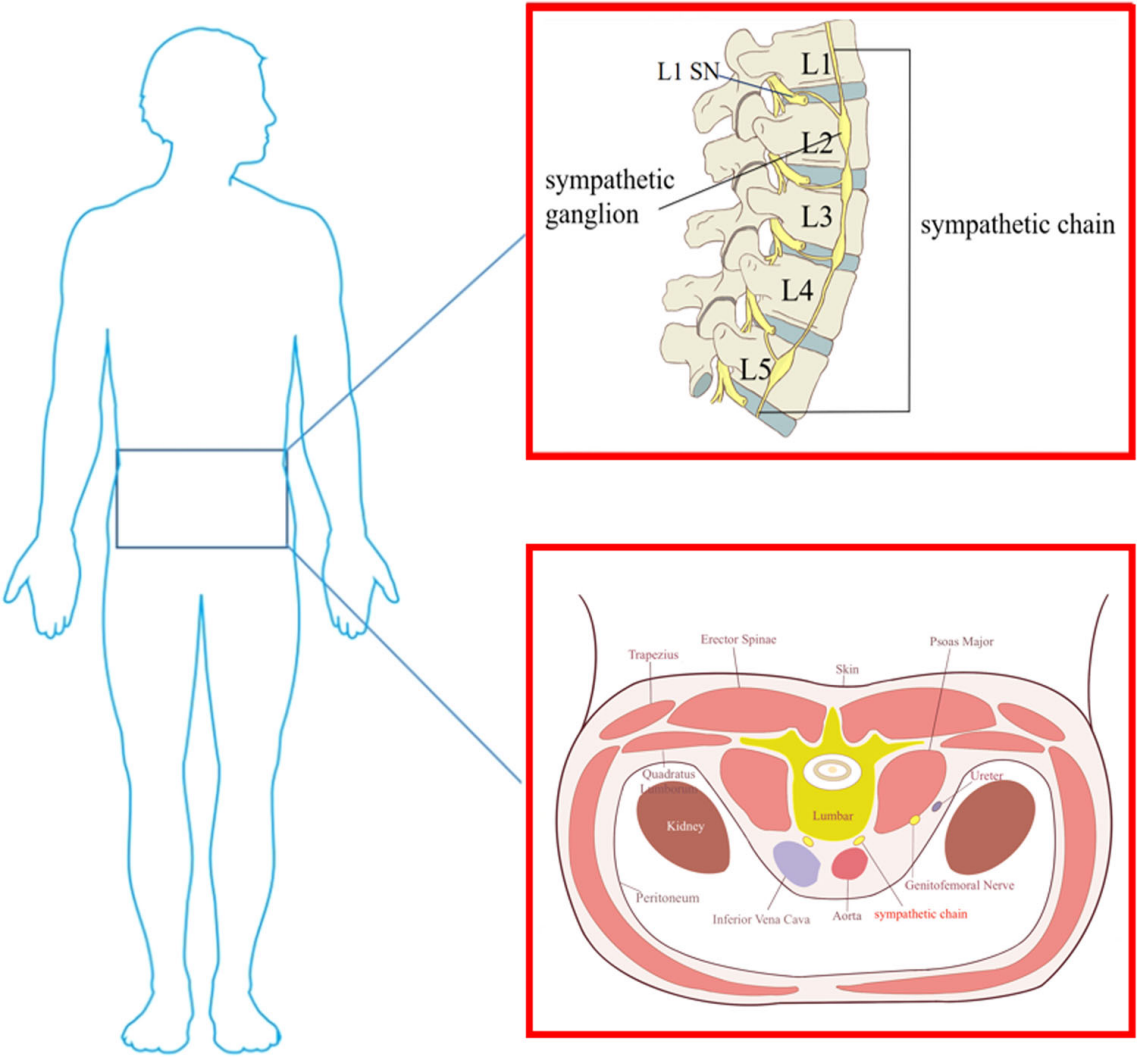
Anatomy of the lumbar sympathetic nerve. SN: sympathetic nerve. L1, L2, L3, L4, L5: lumbar 1, lumbar 2, lumbar 3, lumbar 4, lumbar 5. [Color figure can be viewed at wileyonlinelibrary.com]

The lumbar sympathetic ganglion (LSG) is mostly located at the level of the corresponding vertebral body or between the upper and lower vertebral bodies, usually in the lateral nucleus of the lateral column of the gray matter of the spinal cord at lumbar 3, and their location varies greatly. The preganglionic fibers of the LSN (white communicating branches) originate mainly from the L2‐3 nerve roots. There is no white communicating branch between the sympathetic ganglia below the L3 spinal segment and the corresponding spinal nerves. These sympathetic nerve fibers pass down through the L2 sympathetic ganglion and then return to the lumbar nerve through the gray communicating branch to innervate the lower limbs, and the corresponding nerve areas include the buttocks, sciatic bones, and lower limbs. The densest parts of LSG are located in L2 and L3, and the L2 sympathetic ganglion is mainly located in the lower third (1/3) of the L2 vertebral body, between the L2‐3 intervertebral disc and the upper 1/3 of the L3 vertebral body; the position is relatively fixed.^16^ Therefore, LSGB is most often performed in the lower 1/3 of L2 or the upper 1/3 of L3 (Figure 1).^16^, ^17^

## 3 MECHANISM AND EFFECTS OF LSGB

### 3.1 Denervation of sympathetic effects

LSGB makes sympathetic nerve fibrosis and produces “denervation of sympathetic effect,” which reduces the vascular tension of the lower limbs, relieves vascular smooth muscle spasm, increases the collateral circulation in the skin vascular bed and arteriovenous shunt, and makes the skin of the feet congested and pink color. Previous research has shown that the blockage of sympathetic nerves could reduce the release of plasma endothelin (ET) (which has a strong vasoconstrictor effect and can produce strong arterial spasm), making it and the calcitonin gene‐related peptide (CGRP) (reverse the vasoconstrictive effect of plasma ET, effectively alleviate vasospasm) to achieve dynamic balance. This mechanism can correct the imbalance of vasoactive substance metabolism, further improve lower limb tissue perfusion, promote the elimination of pain‐causing substances, and reduce ischemic pain.^18^, ^19^, ^20^ High‐intensity focused ultrasound (HIFU)‐guided LSG therapy, on the one hand, expands the body's blood vessels, improves local blood supply, reduces inflammatory reactions, and participates in painful nerve endings; on the other hand, it promotes the increase of CGRP and substance P (SP) release from nerve endings, further through positive feedback to promote the release of beta‐endorphin (β‐EP) and terminate the vicious cycle of ischemic pain. At the same time, it can also reduce the SP level in the posterior horn of the spinal cord, and β‐EP gradually returns to a level close to normal.^21^ As a pain transmitter, SP exerts a variety of biological effects, such as causing pain, lowering blood pressure, dilating blood vessels, and increasing capillary permeability.^22^

### 3.2 Sympathetic‐sensory coupling effects

Studies have shown that the pathways of sensory afferent and sympathetic pathways are independent of each other under normal physiological conditions.^23^ However, when peripheral nerves are injured, sympathetic‐sensory coupling occurs in the nerve injury area, tissue inflammation area, noninjured afferent nerve fibers, and dorsal root ganglia (DRG) related to the injury area. In this coupling, α2‐adrenergic receptors in the cell membrane of sensory neurons are upregulated, showing increased sensitivity to adrenergic receptor agonists and abnormal sensitization to post‐sympathetic ganglion fiber excitement, causing hyperalgesia, which triggers pain and spontaneous pain. Iwase et al. demonstrated that sympathetic nerve fibers sprouting in DRG are the main mechanism for the formation of sympathetic‐sensory coupling when peripheral nerves are injured. The sympathetic sprouting branch forms a net‐like structure around sensory neurons to form a connection between the two.^24^ Evidence demonstrated that potassium channel blockers could increase the sprouting of sympathetic postganglionic fibers by promoting the abnormal discharge of sensory neuron terminals.^25^ On the contrary, some studies found that injection of sodium channel blockers into injured nerves can inhibit this spontaneous discharge, reduce sympathetic sprouting, and relieve NP, while injection of normal nerves has no such effect.^25^, ^26^ Moreover, it has been proved that systemic application of triamcinolone acetonide can reduce the gemmation of sympathetic nerve fibers in DRG and reduce the release of cytokines, and it can also relieve pain.^27^

## 4 APPLICATION OF LSGB IN LOWER LIMB PAIN

**Figure 2:**
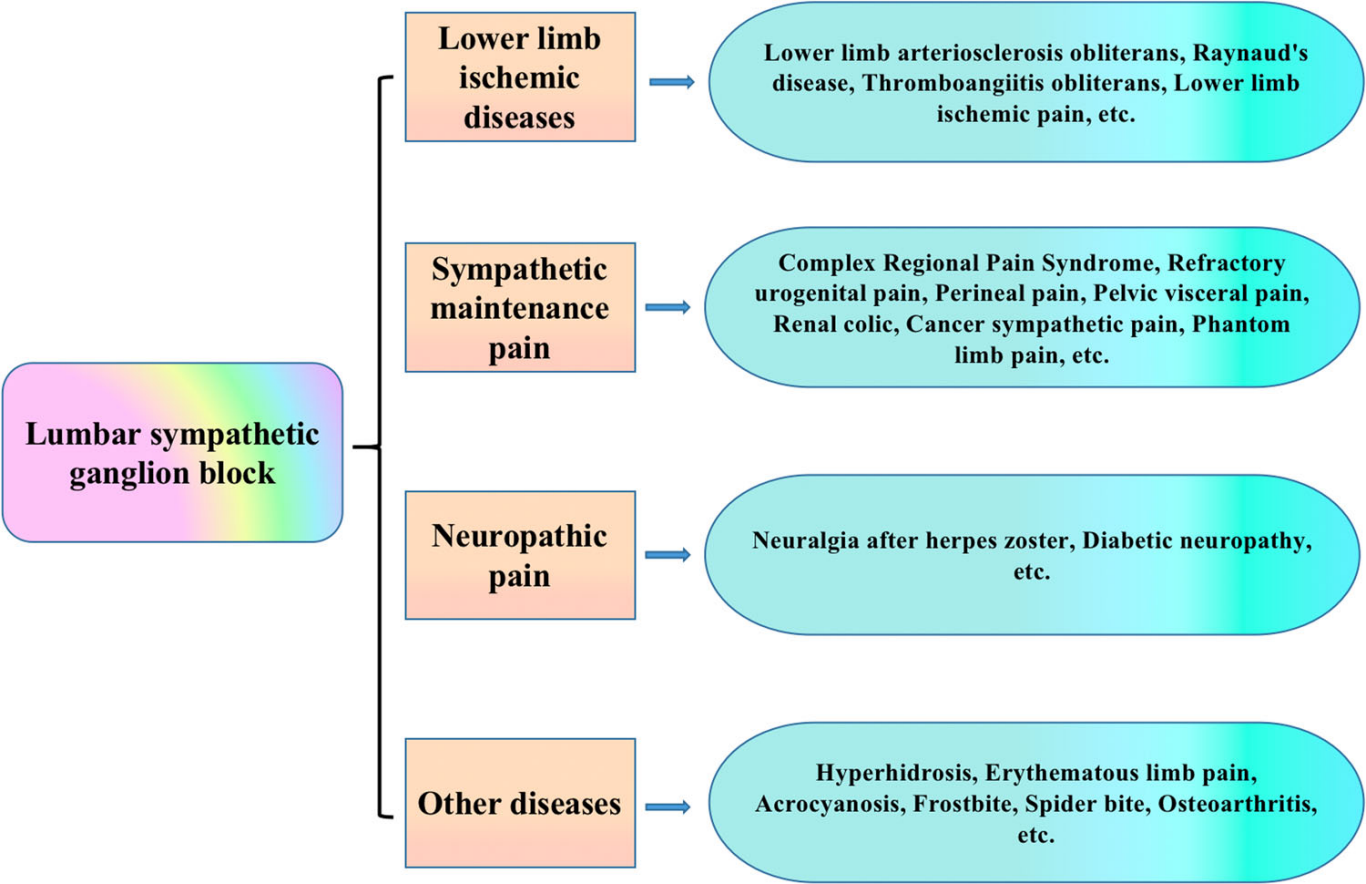
Application of the lumbar sympathetic ganglion block (LSGB) in diseases [Color figure can be viewed at wileyonlinelibrary.com]

LSGB is clinically used for the treatment of a variety of diseases (Figure 2). Here, we focus on the application of LSGB in lower limb pain.

### 4.1 Neuropathic pain

Sympathetic nerves exert a significant signal transmission role in the pathological process of pain, and severing the corresponding sympathetic nerves can result in the disappearance of NP and ischemic pain in the corresponding regions.^28^ Blocking LSN function can achieve continuous vasodilation, improve local blood circulation and nutrient supply, eliminate allodynia, and relieve pain. CRPS is a typical sympathetic maintenance pain, and more and more pieces of evidence showed that patients can get significant pain relief after receiving LSGB.^29^, ^30^, ^31^, ^32^, ^33^, ^34^, ^35^, ^36^, ^37^, ^38^ In addition, postherpetic neuralgia is a common and intractable NP, and its incidence and prevalence gradually increase with age. The study found that patients with postherpetic neuralgia experienced a marked reduction in pain and improvement in quality of life after LSGB.^39^, ^40^ Moreover, diabetic peripheral neuropathy (DPN) and diabetic foot are one of the most common and serious complications of diabetes, respectively, often with severe pain and tissue destruction in the lower extremities, which cannot be ignored. More and more evidence showed that sympathetic nerve blockade for DPN can lastingly provide durable pain relief and increase skin temperature on the affected extremity. LSGB mainly dilates peripheral blood vessels of the lower limb through nerve block, increases blood flow, improves local microcirculation, promotes the establishment of collateral circulation, and plays an important role in preserving limbs.^41^, ^42^ Furthermore, the data show that the morbidity of phantom limb pain (PLP) in amputees ranged from 42% to 78%. Case reports find LSGB is safe and effective for reducing PLP.^43^, ^44^, ^45^ Taken together, LSGB has a significant effect on NP relief (Table 1).^46^, ^47^

**Table 1:** Application summary of the lumbar sympathetic ganglion block (LSGB) in neuropathic pain

Reference	Type of disease	Assistant technology	Puncture location	Drug	Outcomes
[26]	CRPS	Fluoroscopy, ultrasound	L2, L3, L4	Bupivacaine, lidocaine, ropivacaine, medications	39% of respondents perform LSB at the L2, 53% at L3, the remaining at more than one single level (L2‐L4).
[27]	CRPS	No mention	No mention	No mention	Patient received pain relief after intense drugs, LSB, and physical treatment.
[28]	CRPS	Fluoroscopy	L2	1% lidocaine mixed with 100 UI of BTA, 20 ml	After 2 h, the patient was discharged without complaints of pain.
[29]	CRPS	Ultrasound	No mention	1% lidocaine, 20 ml	After two LSGBs, pain scores are decreased and the girl could walk normally.
[30]	CRPS	Fluoroscopy	L2 and L3 level	0.25% ropivacaine mixed with BTA 100 IU or BTB 5000 IU	The VAS scores of patients in the two groups were significantly decreased after LSB, and the LSB effect in the BTB group was longer than that in the BTA group.
[31]	CRPS	Ultrasound	Upper third of the L3 vertebra	10 ml of 0.25% levobupivacaine	A successful case of a US‐guided LSGB without major complications.
[32]	CRPS	No mention	No mention	Lidocaine	The patient showed a marked relief of pain and sensitivity after trial LSB.
[33]	CRPS	Fluoroscopy	L3	0.25% levobupivacaine mixed with BTX‐B 5000 IU, 5 ml	Both pain intensity and LANSS score were observably decreased after LSB with BTX‐B; Besides, Skin color returned to normal, allergies and cold sensations disappeared.
[34]	CRPS	Fluoroscopy	L4	2% lidocaine and 0.5% bupivacaine (1:1), 15 ml	Patient's lower extremity temperatures were noted to be 24°C bilaterally at the feet and 27.8°C at the ankles. Her pain level was 0/10.
[35]	CRPS	CT	L3	0.5% bupivacaine, 4 ml, ethanol 96%, 2 ml	The patient's NRS score decreased from 7 to 4, and the skin temperature of the right foot increased significantly.
[36]	Intractable lower‐limb PHN	Fluoroscopy	L3	0.5% bupivacaine mixed with 40 mg of triamcinolone	LSB relieved the pain of two cases of lower extremity systemic drug‐resistant PHN, and NRS scores decreased by at least 50%.
[37]	Postherpetic neuralgia secondary to zoster	Full text is unavailable	Full text is unavailable	Full text is unavailable	After LSGB, patients who suffered postherpetic neuralgia secondary to zoster recieved pain relief and improvement in quality of life.
[38]	Refractory diabetic neuropathy	CT	Standard posterolateral approach	1% lidocaine mixed with the iohexol mixture/lidocaine mixed with dexamethasone, 20 ml	After treatment, the VAS pain scores of each patient were dramatically reduced; the blood oxygen saturation, capillary refill time, and skin temperature were also markedly ameliorated in patients.
[39]	Refractory painful diabetic neuropathy	Fluoroscopy	L3	1% lidocaine mixed with 20 mg of Triamcinolone, 12 ml	The NRS scores were markedly decreased, and the temperature was strikingly increased after the treatment.
[40]	Postamputation pain	Fluoroscopy	L2	0.25% bupivacaine, 10 ml	Patients with PAP who received a single LSG could reduce both pain and PDI.
[43]	Anterior lumbar interbody fusion (ALIF)	Fluoroscopy	L3	0.36% ropivacaine, 10 ml	The patient's NRS decreased from 9 to 4 after treatment, and the ipsilateral plantar temperature increased by more than 2°C.
[44]	NP of the lower limb	Fluoroscopy	The lower 1/3 of the L2 or the upper 1/3 of the L3	1% lidocaine, 8–10 ml	PTT detection was an early objective index to judge the success of LSGB.

Abbreviations: BTA/BTB, botulinum toxin type A/B; CRPS, complex regional pain syndrome; LANSS, leeds assessment of neuropathic symptoms and signs; LSB/LSGB, lumbar sympathetic block/lumbar sympathetic ganglion block; NP, neuropathic pain; NRS, numeric rating scales; PAP, postamputation pain; PDI, pain disability index; PHN, post herpetic neuralgia; PTT, pulse transit time.

### 4.2 Lower limb ischemic pain

Studies have found that around 20% of patients who suffered lower limb ischemic pain are not suitable for surgical intervention for various reasons. In these patients, LSGB can be used to reduce pain, improve the walking status and activities of daily living, and may delay or avoid amputation. The use of LSGB can destroy the innervation of sympathetic nerves on the blood vessels of the lower extremities, and the innervated blood vessels continue to expand to improve local blood circulation and nutrient supply, thereby reducing pain (Table 2).^47^, ^48^, ^49^, ^50^, ^51^

**Table 2:** Application summary of the lumbar sympathetic ganglion block (LSGB) in lower limb ischemic pain

Reference	Type of disease	Assistant technology	Puncture location	Drug	Outcomes
[45]	Peripheral arterial disease	Fluoroscopy	L2, L3, and L4	1% lidocaine and radiographic dye (1:1), 2 ml	Increased BSSP after LSB was assessed by LSFG, indicating improved foot circulation.
[46]	Peripheral arterial disease	Ultrasound	L3 vertebral level	0.2% ropivacaine with 15‐mcg clonidine, 20 ml	NRS dropped below 3 after ULSB. All patients had a 2°C rise in body temperature from baseline with high satisfaction.
[47]	Chronic obstructive arterial disease	Fluoroscopy	L2‐L3‐L4	0.25% bupivacaine with absolute alcohol, 3 ml	LSB is safe and effective for pain relief in patients with critical limb ischemia.
[48]	Livedo reticularis	Fluoroscopy	L3, L4	Total phenol amount for both sides of <10 ml	The postoperative skin surface temperature increases.

Abbreviations: BSSP, beat strength of skin perfusion; LSB, lumbar sympathetic block; LSFG, laser speckle flowgraphy; ULSB: ultrasound‐guided lumbar sympathetic block.

## 5 POTENTIAL COMPLICATIONS OF LSGB

The most common acute complications of LSGB are local hematoma and local pain (at the injection site), which usually resolve spontaneously within a few hours to a few days. In addition, some studies have reported dizziness, headache, and weakness or pain in the injected leg that is possibly due to damage to the genital femoral and lateral femoral cutaneous nerves.^38^, ^52^, ^53^, ^54^ Moreover, there are also some serious complications, such as accidentally stabbed into the subarachnoid space and epidural causing extensive blockade after injection and resulting in respiratory and circulatory disorders, nerve injury by repeated puncture, blood vessels or the lumbar intervertebral disc damage, intravascular or intralymphatic injection, infection, ureteral or renal injury, drug hypersensitivity reactions, and local anesthetic toxicity, although these are extremely rare.^55^, ^56^, ^57^, ^58^

## 6 ADVANTAGES OF ULTRASOUND APPLICATION IN LSGB

Because the LSN is adjacent to important large blood vessels, spinal cord, and nerves, this operation must be performed by a physician who is very familiar with the local anatomy and has experience in the treatment with the aid of positioning. LSGB is usually implemented with the assistance of fluoroscopy and computed tomography (CT). Nevertheless, when performing fluoroscopy and CT, the assistance of an equipped operating room or radiology department is required, and the level of radiation exposure is higher with repeated use.^59^ Ultrasound‐guided techniques, introduced into pain treatment in the mid‐2000s, have been proven to be involved in a number of advantages, such as the ability to imagine the structures of soft tissues, watch diffusion patterns in real time for needle insertion, and drug injection with minimal radiation exposure.^60^, ^61^, ^62^ Based on the ability of ultrasound‐guided LSGB to observe and identify tissue structures, the route of puncture needles, and the diffusion of local anesthetics in real‐time, it can improve the success rate of puncture, reduce complications, and avoid radiation damage, with obvious clinical advantages.

## 7 APPLICATION PROSPECT OF LSGB DURING THE PERIOPERATIVE PERIOD

**Figure 3:**
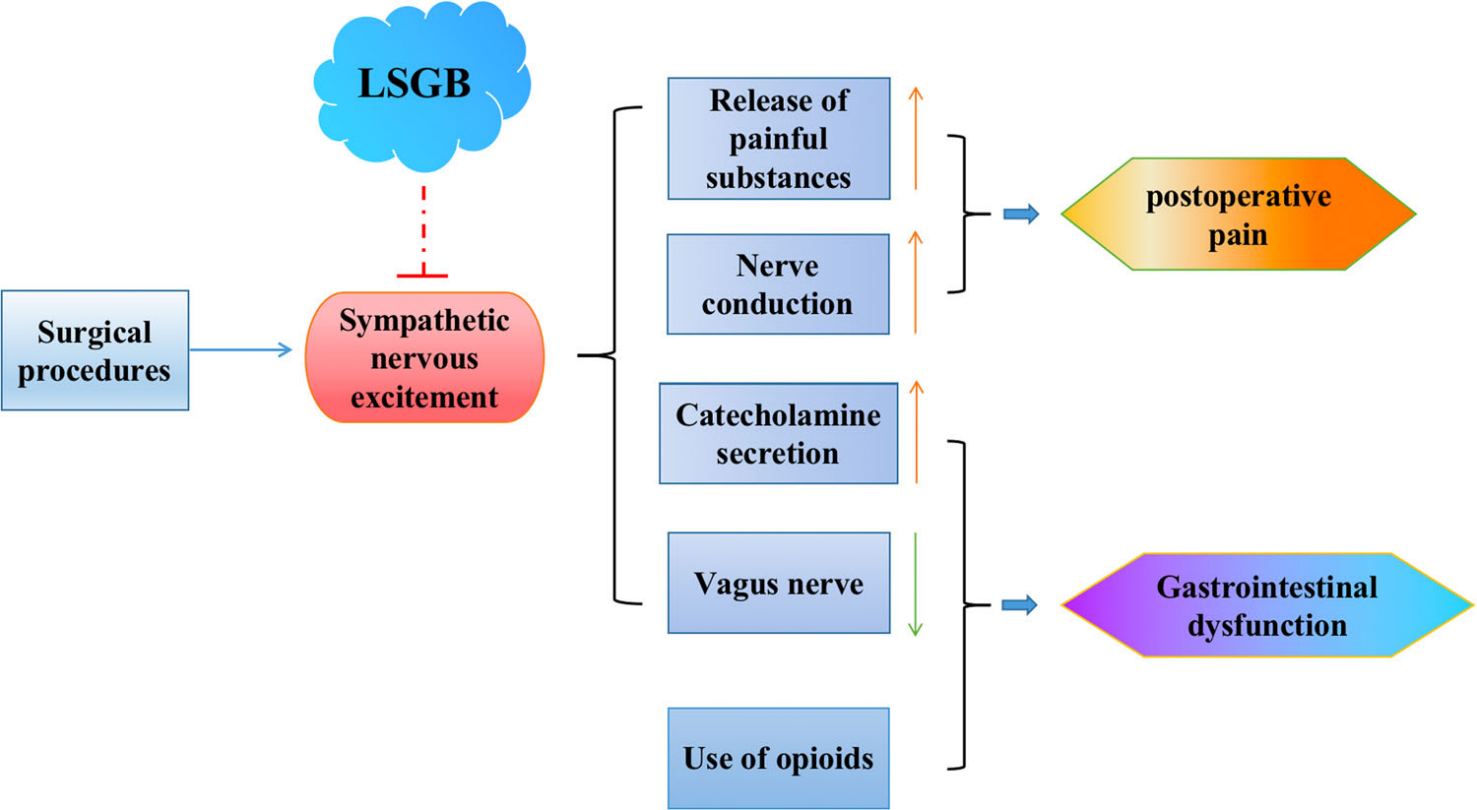
Application prospect of the lumbar sympathetic ganglion block (LSGB) during the perioperative period [Color figure can be viewed at wileyonlinelibrary.com]

LSN is adjacent to a number of vital organs and a large number of large blood vessels, and there is a risk of injury to the large vessels or puncture of the lumbar intervertebral discs by blind puncture. An ultrasound‐guided nerve block can directly observe the relationship between the needle and nerve and surrounding vascular tissues and can see the entire dynamic process of local diffusion after drug injection, which not only shortens the operation time and increases the success rate of the block, but also reduces the dosage of local anesthetics, the toxicity of the drug, and the risk of complications. Moreover, LSGB has a good application prospect during the perioperative period (Figure 3).

### 7.1 LSGB application in perioperative analgesia

Chronic pain has been shown to be sympathetically related.^10^ LSGB is widely used to diagnose and treat persistent sympathetic pain.^9^, ^10^ Under ultrasound guidance, a local anesthetic can be accurately injected, and blocking LSG can effectively block sensation in the corresponding area. Studies have reported that patients with cancer‐related pain in the back, abdomen, pelvis, or legs can have their pain reduced by LSGB. LSGB provides good analgesia after surgery.^63^ With regard to perioperative pain management, LSGB can reduce residual limb pain and PLP during the perioperative period and help patients to recover psychologically after surgery.^43^ The causes of pain after lumbar spine surgery are complex. Changes in the autonomic nervous system (ANS) have been reported to exert a vital role in the generation and maintenance of pain in patients who suffered from failed back surgery syndrome (FBSS). While LSGB has achieved good efficacy in pain relief of patients who suffered FBSS.^64^ Severe sympathetic persistent pain can occur after anterior spinal surgery, and the application of LSGB can successfully relieve pain.^45^

In recent years, LSGB has attracted the attention of anesthesiologists and microsurgeon in skin flap transplantation due to its good analgesic effect and unsympathetic vasoconstrictor effect, which can dilate blood vessels, improve blood supply to the lower limb, and promote the establishment of collateral circulation.^7^, ^65^ For lower limb flap transplantation, an intrathecal block is widely used. However, spinal anesthesia blocks the sympathetic activity and also blocks the motor function of the lower limb, which causes inconvenience for patients to move after surgery, increases the risk of venous thrombosis of the lower extremities, and is not conducive to rapid perioperative recovery.^66^, ^67^ Studies have shown that the introduction of LSGB for perioperative analgesia in lower limb free flap transplantation can reduce perioperative pain, increase the temperature of the transplanted flap, improve the blood supply of the transplanted flap, and promote the survival of the transplanted flap. In turn, it can improve sensory function recovery in patients with transplanted skin flaps, resulting in clinical benefits in patients undergoing lower limb free flap transplantation.^7^, ^34^, ^68^

### 7.2 Application prospect of LSGB in the regulation of gastrointestinal function

It is well known that the stimulation of the surgical operation can increase the excitability of the sympathetic nerves, which will lead to a significant increase in the postoperative plasma catecholamine level. This in turn leads to a high degree of the mesenteric blood vessels and a reduction in the blood supply of the gastrointestinal tract, ultimately resulting in the long‐term inhibition of gastrointestinal motility. In addition, it can also reflexively inhibit the efferent fibers of the vagus nerve and weaken the motor function of the gastrointestinal tract. Meanwhile, because the inhibition of the vagus nerve blocks the body's cholinergic anti‐inflammatory pathway, the effect of inhibiting the secretion of inflammatory cytokines is weakened or disappeared, resulting in gastrointestinal dysfunction.^69^, ^70^ In addition, the use of opioids during anesthesia and postoperative analgesia inhibits bowel motility and delays the recovery of gastrointestinal function. Research has shown that postoperative ileus duration was positively correlated with perioperative opioid consumption. Moreover, different anesthesia methods have different effects on inhibiting postoperative gastrointestinal motility, among which general anesthesia has the greatest effect.^71^ Studies have shown that the rehabilitation time of postoperative gastrointestinal function with abdominal surgery under general anesthesia combined with epidural anesthesia is significantly shorter than that in patients with traditional general anesthesia.^72^

Importantly, LSGB in surgery can reduce the use of anesthetics, inhibit the fluctuation of hemodynamics, have a positive auxiliary effect on patients with cardiopulmonary insufficiency, and can effectively ameliorate the quality of analgesia for patients. LSGB can produce a good postoperative analgesic effect, reduce postoperative pain, effectively shorten the recovery time of patients, and improve patient satisfaction after the operation. In addition, the postganglionic fibers of LSN directly innervate internal organs, glands, and visceral blood vessels.^73^ LSGB can inhibit sympathetic nerve activity, dilate blood vessels, and block the stress response caused by external stimuli, which is conducive to reducing postoperative gastric intestinal dysfunction. Thus, the above description indicates that LSGB has potential application value in the regulation of gastrointestinal function during the perioperative period. However, there is currently a lack of evidence for the study of gastrointestinal function in LSGB during the perioperative period. Therefore, this review is just theoretical speculation, and the feasibility still needs more clinical trials and data to prove.

## 8 CONCLUSION

Overall, LSGB is an effective treatment for patients with lower limb pain and postoperative pain and has great application prospects in the recovery of gastrointestinal function after surgery. Further clinical trials are needed to confirm its efficacy during the perioperative period.

## AUTHOR CONTRIBUTIONS

Min‐Jian Geng conceived the study, wrote, and revised the manuscript; Jing‐Han Zhang and Yan‐Ping Deng collected the literature and summarized the data.

## CONFLICT OF INTEREST

The authors declare no conflict of interest.

## ETHICS STATEMENT

Not applicable.

## Data Availability

The data contained in the article can be reasonably requested from the corresponding author.
